# Snap Diagnosis: Developing an Artificial Intelligence Algorithm for Penile Cancer Detection from Photographs

**DOI:** 10.3390/cancers16233971

**Published:** 2024-11-27

**Authors:** Jianliang Liu, Jonathan S. O’Brien, Kishor Nandakishor, Niranjan J. Sathianathen, Jiasian Teh, Todd Manning, Dixon T. S. Woon, Declan G. Murphy, Damien Bolton, Justin Chee, Marimuthu Palaniswami, Nathan Lawrentschuk

**Affiliations:** 1 EJ Whitten Prostate Cancer Research Centre, Epworth Healthcare, Melbourne, VIC 3005, Australia; 2 Department of Urology, The Royal Melbourne Hospital, The University of Melbourne, Melbourne, VIC 3052, Australia; 3 Department of Surgery, University of Melbourne, Melbourne, VIC 3052, Australia; 4 Sir Peter MacCallum Department of Genitourinary Oncology, The University of Melbourne, Melbourne, VIC 3052, Australia; 5 Department of Electrical and Electronic Engineering, The University of Melbourne, Parkville, VIC 3052, Australia; 6 Department of Surgery, Austin Health, The University of Melbourne, Melbourne, VIC 3052, Australia; 7 MURAC Health, East Melbourne, VIC 3002, Australia

**Keywords:** artificial intelligence (AI), deep learning (DL), diagnosis, early detection of cancer, health information technology, neural networks (CNN), penile neoplasm, penile carcinoma in situ (CIS), penile intraepithelial neoplasia

## Abstract

**Background/Objective**: Penile cancer is aggressive and rapidly progressive. Early recognition is paramount for overall survival. However, many men delay presentation due to a lack of awareness and social stigma. This pilot study aims to develop a convolutional neural network (CNN) model to differentiate penile cancer from precancerous and benign penile lesions. **Methods:** The CNN was developed using 136 penile lesion images sourced from peer-reviewed open access publications. These images included 65 penile squamous cell carcinoma (SCC), 44 precancerous lesions, and 27 benign lesions. The dataset was partitioned using a stratified split into training (64%), validation (16%), and test (20%) sets. The model was evaluated using ten trials of 10-fold internal cross-validation to ensure robust performance assessment. **Results:** When distinguishing between benign penile lesions and penile SCC, the CNN achieved an Area Under the Receiver Operating Characteristic Curve (AUROC) of 0.94, with a sensitivity of 0.82, specificity of 0.87, positive predictive value of 0.95, and negative predictive value of 0.72. The CNN showed reduced discriminative capability in differentiating precancerous lesions from penile SCC, with an AUROC of 0.74, sensitivity of 0.75, specificity of 0.65, PPV of 0.45, and NPV of 0.88. **Conclusion:** These findings demonstrate the potential of artificial intelligence in identifying penile SCC. Limitations of this study include the small sample size and reliance on photographs from publications. Further refinement and validation of the CNN using real-life data are needed.

## Simple Summary

Penile cancer is aggressive and rapidly progressive. Early detection is crucial for survival. Many men delay seeking help due to a lack of awareness and fear of embarrassment. This study explored the use of artificial intelligence (AI) in detection of penile cancer. AI was trained on 136 penile lesions images from scientific publications. This included 65 penile cancer, 44 precancerous, and 27 benign images. It performed well in distinguishing between benign lesions and penile cancer with high accuracy. However, it faced challenges in accurately identifying precancerous lesions. These findings suggest that AI has potential in early detection of penile cancer, but more research is needed to refine and validate the AI software with real-life data. The goal is to complement and not replace clinicians. The AI algorithm may allow patients to evaluate any concerning penile lesion from the comfort and privacy of their home, potentially encouraging earlier medical consultation.

## 1. Introduction

In developed countries, penile cancer occurs in up to 1 in 100,000 men [1,2,3]. The prevalence increases up to 7 in 100,000 men in certain regions of Africa, Asia, and South America [1,4]. Although relatively uncommon, it presents a significant issue due to its aggressive nature with early lymphatic spread and a lack of sensitive imaging for the detection of micrometastatic disease [1,5]. Early recognition of penile cancer prior to lymphatic involvement is paramount for the patient’s overall survival [6]. Additionally, early detection allows for organ-preserving penile surgery [7]. This includes partial resection with possible reconstruction, laser ablation, and topical therapy [8,9]. These organ-preserving penile procedures may allow for better outcomes in terms of sexual function, urinary function, and quality of life [10,11]. However, many men have delayed presentation due to a lack of awareness, social stigma, fear of embarrassment, and limited access to culturally appropriate care [12]. Public health campaigns aimed at increasing awareness, promoting better hygiene, and deploying human papillomavirus (HPV) vaccines have had varied success in decreasing the disease burden [2]. There is an urgent need to improve the early detection of penile cancer and reduce barriers to subspecialist penile cancer care.

The global healthcare community has begun leveraging artificial intelligence (AI) to address many challenges in diagnostic medicine, and its application in the field of oncology has gained significant momentum [13,14]. The strengths of AI lie in its ability to analyse large datasets, account for multiple variables, and identify subtle patterns that may not be evident to the human eye [15]. Particularly, the use of convolutional neural network (CNN) to process photographs of skin cancers through layers of interconnected artificial neural networks that make hierarchical decisions mimicking the neurons and decision-making process of the brain [16].

These advancements have contributed to breakthroughs in fields like dermatology, where AI algorithms are increasingly being used to detect skin cancers such as melanoma and squamous cell carcinoma (SCC) [17]. A systematic review of 272 studies using AI to evaluate skin cancer in a primary care setting demonstrated a mean diagnostic accuracy of 89.5% (range 59.7–100%) for melanoma and accuracy of 85.3% (range 71.0–97.8%) for SCC [18]. In another systematic review of 53 studies, AI’s performance (sensitivity of 86.3% and specificity of 78.4%) was comparable to the diagnostic capabilities of expert clinicians (sensitivity of 84.2% and specificity of 74.4%) [19]. When evaluating the role of deep learning in diagnosing melanoma on dermatoscopic images, 37 studies demonstrated a pooled area under the curve (AUC) of 0.87 (range 0.84–0.90), which was comparable to the performance of dermatologists who had a pooled AUC of 0.83 (range 0.79–0.86) [20].

Penile cancer often manifests in its early stages with non-specific symptoms such as erythema, plaque formation, or ulceration, complicating early diagnosis [21]. With 95% of penile cancers being SCC, these lesions exhibit macroscopic similarities to other cutaneous SCC [22]. Despite advancements in AI for skin cancer detection, there is currently no AI algorithm for the assessment of penile lesions. This pilot study aims to evaluate the potential of AI in differentiating between various types of penile lesions such as benign, pre-cancerous, and penile SCC.

## 2. Materials and Methods

**Figure 1:**
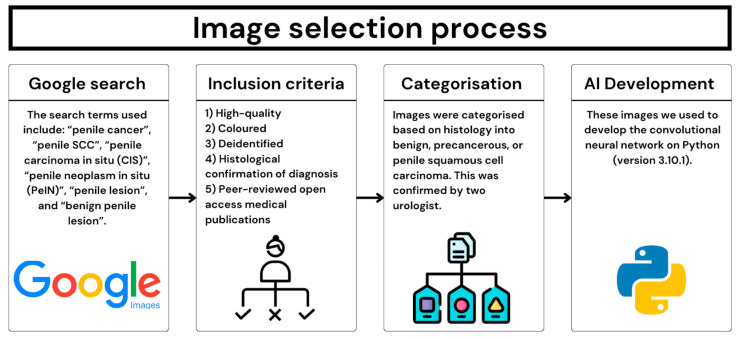
Selection process of included images.

A search was performed on Google Images (Alphabet Inc., Mountain View, CA, USA) to identify images of penile lesions from peer-reviewed open access medical publications. The search terms used include: “penile cancer, “penile SCC”, “penile carcinoma in situ (CIS)”, “penile neoplasm in situ (PeIN)”, “penile lesion”, and “benign penile lesion”. The inclusion criteria are that the images had to be high-quality, coloured, deidentified, and focusing only on the penile lesion (See Figure 1). Images were excluded if they were poor-quality (such as pixelated) or if there was no histological confirmation of diagnosis. The images were then categorised and labelled into benign, precancerous, or penile SCC. Pre-cancerous penile lesions were defined as Bowen’s Disease, PeIN, and CIS. For the images to be included as precancerous or penile SCC, the penile lesion needed to have a histologically confirmed diagnosis. Two urologists who specialise in the management of penile cancer, independently reviewed the images to ensure they met the inclusion criteria and that the images had been appropriately labelled and categorised. These final included images from the dataset.

**Figure 2:**
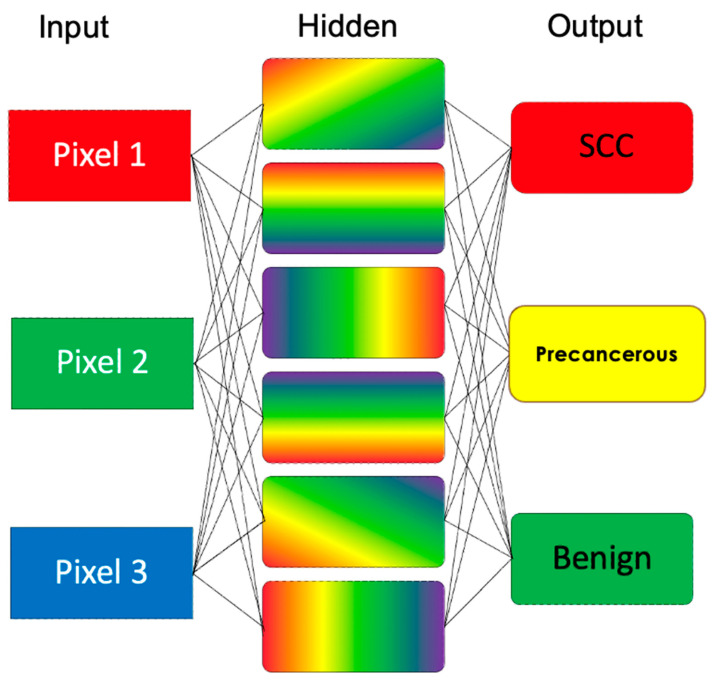
Model of convolutional neural network used.

Python (version 3.10.1, Python Software Foundation, Wilmington, DE, USA) was used to develop the CNN-based algorithm and to evaluate diagnostic performance (Area Under the Receiver Operating Characteristic Curve (AUROC), sensitivity, specificity, Positive Predictive Value (PPV), and Negative Predictive Value (NPV)). The CNN automatically extracts and segments the image into pixels in the input layer (see Figure 2). In the hidden layer, the CNN utilises pixel edge contours to generate a “mask” representation of the lesion against normal skin, capturing features such as lesion elevation, erythema, ulceration, redness, and irregularity. Internal validation was performed to evaluate the algorithm’s accuracy in lesion classification. The output was to determine if the penile lesion was benign, precancerous, or penile SCC. No patient identifiers, demographics, or risk factors were used in the development of the CNN.

**Figure 3:**
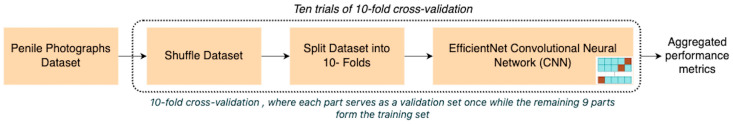
Flow diagram of the convolutional neural network.

A CNN-based architecture that excels in image classification tasks was used, as it is capable of efficiently scaling up in terms of layer depth, layer width, input resolution, or a combination of these factors (see Figure 3). This model involves balancing network depth, width, and resolution to achieve optimal performance. The model can efficiently scale up in complexity while maintaining accuracy. Meta-analyses of dermatological imaging applications have established CNNs as the predominant deep-learning approach, consistently demonstrating superior diagnostic performance in skin lesion classification tasks [16]. CNNs are highly effective for image tasks due to their ability to capture spatial hierarchies and patterns in visual data through convolutional layers, enabling automatic feature learning at multiple scales without manual feature engineering. The Standardised Reporting of Machine Learning Applications in Urology (STREAM-URO) framework was employed to ensure the quality of this study [23].

## 3. Results

One hundred thirty-six images from 83 articles were included in this study. This comprised 65 penile SCC, 44 precancerous penile lesions, and 27 benign penile lesions. The image samples were divided into training (64%), validation (16%), and test (20%) subsets. The training subset was utilised to train the CNN architecture, the validation subset assisted in selecting a model with good generalisation performance, and the independent test subset provided an unbiased evaluation of the model’s final performance metrics, ensuring robust assessment of generalizability to unseen data.

We performed ten trials of 10-fold internal cross-validation on the dataset of 136 images. The dataset was divided into ten folds, each containing penile SCC images and precancerous or benign images of penile lesions. Nine of the ten folds were used for training the CNN, while one fold was used for testing the algorithm. This process was repeated for ten rounds of validation, resulting in a total of 100 experiments.

The diagnostic performance of the CNN for distinguishing between benign penile lesions and penile SCC was as follows: AUROC of 0.94, sensitivity of 0.82, specificity of 0.87, PPV of 0.95, and NPV of 0.72 (see Table 1). When differentiating between precancerous lesions from penile SCC, the CNN obtained an AUROC of 0.74, sensitivity of 0.75, specificity of 0.65, PPV of 0.45, and NPV of 0.88. The algorithm demonstrated an overall triage accuracy of 87.5%.

## 4. Discussion

Multiple benign and infectious conditions can result in penile lesions, which may cause unnecessary distress to patients who mistakenly perceive them as penile cancer [24,25]. The findings from this study highlight the potential of CNNs in aiding the differentiation of benign penile lesions from penile SCC. The CNN demonstrated high diagnostic accuracy with an AUROC of 0.94 when distinguishing benign penile lesions and penile SCC. This indicates that the CNN effectively utilises visual features extracted from images to accurately classify these lesions. Moreover, the AI model demonstrated a robust sensitivity of 0.82 and a specificity of 0.87, highlighting its ability to reliably detect malignant lesions while minimising false positives, thus providing a valuable diagnostic tool in clinical settings.

However, the performance of the CNN appeared to be less robust when differentiating precancerous lesions from penile SCC, as indicated by a lower AUROC of 0.74, sensitivity of 0.75, and specificity of 0.65 in this subgroup analysis. One hypothesis for this discrepancy could be the subtle visual distinctions between precancerous changes and early superficial penile SCC, which may pose challenges even to trained eyes [26]. We are hopeful that future iterations of the AI, trained on larger datasets, will have an improved ability to distinguish precancerous lesions from penile SCC. The purpose of the future AI-powered smartphone application is not to replace the need for clinician review nor to give an accurate diagnosis. The main goal of our future smartphone application is to give an estimated likelihood of penile cancer, to encourage patients to present to their doctors and educate them about the aggressive nature of penile cancer. Consequently, rather than simply distinguishing between precancerous and cancerous lesions, which both require treatment, the ability to differentiate benign penile lesions will be one of the application’s most valuable features.

Another potential application of this technology is in the primary care setting, where general practitioners could use the CNN to confirm the absence of malignancy in suspected benign lesions. This hypothesis is supported by a previous systematic review which demonstrated that AI could aid in the early detection of skin cancer in community and primary care settings with high accuracies [22]. Although a similar challenge may be faced in terms of differentiating between precancerous lesions and penile SCC, it does not alter management in a primary care setting, as both types of lesions require referral to urologists specialised in penile cancer.

Menzies et al. conducted a multicentre, prospective clinical trial to evaluate an AI-powered smartphone application for skin cancer diagnosis in the secondary care setting [27]. Although the AI algorithm previously outperformed specialists in an experimental online reader study, it was inferior to specialist diagnosis in the real-world trial [27]. Therefore, emphasising the necessity for future penile cancer-related AI applications to undergo rigorous real-world testing. Interestingly, in the same trial, the AI software outperformed junior doctors and dermatology trainees.

We appreciate that there are limitations to this study. Firstly, the sample size is relatively small. Secondly, using of images sourced from peer-reviewed journals may differ from real-life photographs taken in clinical settings in terms of quality and presentation, potentially affecting CNN’s performance in real-world applications. We also acknowledge that there are other subtypes of penile cancer other than SCC. To avoid any information that may be used to trace back to the patients, the CNN does not consider age or risk factors such as HPV [2].

Future research directions will focus on developing and validating a CNN architecture using a prospective cohort of histopathologically confirmed penile squamous cell carcinoma (SCC), precancerous lesions, and benign conditions captured in clinical settings. The CNN’s clinical utility will be evaluated through prospective multicentre validation trials, following established methodological frameworks for artificial intelligence in medical imaging similar to Menzies et al. [27]. Upon robust validation, the model will be integrated into a mobile health application, designed to facilitate patient-initiated lesion assessment in a privacy-preserving environment. The platform will serve as a complementary diagnostic aid to primary care assessment, potentially addressing current barriers in early detection while maintaining appropriate clinical oversight.

We acknowledge that multiple ethical and legal considerations are involved in developing such technologies [28]. We are committed to adhering to guidelines like those provided by the European Academy of Dermatology and Venereology (EADV) [29]. One of the anticipated challenges is the acquisition of an adequate volume of images to effectively train the CNN, given the rarity of penile cancer. Collaboration with international cancer societies and urology associations will be crucial in expanding our image database to include diverse ethnicities and geographic regions. This collaborative effort enriches the CNN’s training dataset and enhances its ability to accurately diagnose penile lesions across varied demographics.

## 5. Conclusions

In conclusion, this pilot study shows that CNNs can effectively distinguish benign from malignant penile lesions with encouraging accuracy. However, further refinement is necessary to better differentiate precancerous from malignant lesions. These results highlight AI’s potential to support risk stratification in penile lesions, streamlining the triage process and enabling earlier medical attention.

## Figures and Tables

**Table 1:** Diagnostic performance of the convolutional neural network.

Comparators	AUROC	Sensitivity	Specificity	PPV	NPV
Benign versus penile SCC	0.94	0.82	0.87	0.95	0.72
Precancerous versus penile SCC	0.74	0.75	0.65	0.45	0.88

Abbreviations: Area Under the Receiver Operating Characteristic Curve (AUROC), Positive Predictive Value (PPV), Negative Predictive Value (NPV), Squamous cell carcinoma (SCC).

## Data Availability

No new data were created.

## References

[1] Thomas A., Necchi A., Muneer A., Tobias-Machado M., Tran A.T.H., Van Rompuy A.-S., Spiess P.E., Albersen M. (2021). Penile cancer. Nat. Rev. Dis. Primers.

[2] Douglawi A., Masterson T.A. (2019). Penile cancer epidemiology and risk factors: A contemporary review. Curr Opin Urol..

[3] Douglawi A., Masterson T.A. (2017). Updates on the epidemiology and risk factors for penile cancer. Transl. Androl. Urol..

[4] Fu L., Tian T., Yao K., Chen X.F., Luo G., Gao Y., Lin Y.F., Wang B., Sun Y., Zheng W. (2022). Global Pattern and Trends in Penile Cancer Incidence: Population-Based Study. JMIR Public Health Surveill..

[5] Pow-Sang M.R., Ferreira U., Pow-Sang J.M., Nardi A.C., Destefano V. (2010). Epidemiology and natural history of penile cancer. Urology.

[6] Ficarra V., Akduman B., Bouchot O., Palou J., Tobias-Machado M. (2010). Prognostic factors in penile cancer. Urology.

[7] Burnett A.L. (2016). Penile preserving and reconstructive surgery in the management of penile cancer. Nat. Rev. Urol..

[8] Yuvaraja T.B., Waigankar S., Dharmadhikari N., Pednekar A. (2017). Organ Preservation Surgery for Carcinoma Penis. Indian J. Surg. Oncol..

[9] Zekan D., Praetzel R., Luchey A., Hajiran A. (2024). Local Therapy and Reconstruction in Penile Cancer: A Review. Cancers.

[10] Yao H.H., Sengupta S., Chee J. (2020). Penile sparing therapy for penile cancer. Transl. Androl. Urol..

[11] Falcone M., Preto M., Gül M., Şahin A., Scavone M., Cirigliano L., Peretti F., Ferro I., Plamadeala N., Gontero P. (2024). Functional outcomes of organ sparing surgery for penile cancer confined to glans and premalignant lesions. Int. J. Impot. Res..

[12] Skeppner E., Andersson S.O., Johansson J.E., Windahl T. (2012). Initial symptoms and delay in patients with penile carcinoma. Scand. J. Urol. Nephrol..

[13] Abbas S., Asif M., Rehman A., Alharbi M., Khan M.A., Elmitwally N. (2024). Emerging research trends in artificial intelligence for cancer diagnostic systems: A comprehensive review. Heliyon.

[14] Bhinder B., Gilvary C., Madhukar N.S., Elemento O. (2021). Artificial Intelligence in Cancer Research and Precision Medicine. Cancer Discov..

[15] Davenport T., Kalakota R. (2019). The potential for artificial intelligence in healthcare. Future Healthc. J..

[16] Lyakhova U.A., Lyakhov P.A. (2024). Systematic review of approaches to detection and classification of skin cancer using artificial intelligence: Development and prospects. Comput. Biol. Med..

[17] Melarkode N., Srinivasan K., Qaisar S.M., Plawiak P. (2023). AI-Powered Diagnosis of Skin Cancer: A Contemporary Review, Open Challenges and Future Research Directions. Cancers.

[18] Jones O.T., Matin R.N., van der Schaar M., Prathivadi Bhayankaram K., Ranmuthu C.K.I., Islam M.S., Behiyat D., Boscott R., Calanzani N., Emery J. (2022). Artificial intelligence and machine learning algorithms for early detection of skin cancer in community and primary care settings: A systematic review. Lancet Digit. Health.

[19] Salinas M.P., Sepúlveda J., Hidalgo L., Peirano D., Morel M., Uribe P., Rotemberg V., Briones J., Mery D., Navarrete-Dechent C. (2024). A systematic review and meta-analysis of artificial intelligence versus clinicians for skin cancer diagnosis. NPJ Digit Med..

[20] Ye Z., Zhang D., Zhao Y., Chen M., Wang H., Seery S., Qu Y., Xue P., Jiang Y. (2024). Deep learning algorithms for melanoma detection using dermoscopic images: A systematic review and meta-analysis. Artif. Intell. Med..

[21] Engelsgjerd J.S., Leslie S.W., LaGrange C.A. (2024). Penile Cancer and Penile Intraepithelial Neoplasia.

[22] Cancer Council Australia: Penile Cancer. https://www.cancer.org.au/cancer-information/types-of-cancer/rare-cancers/penile-cancer.

[23] Kwong J.C.C., McLoughlin L.C., Haider M., Goldenberg M.G., Erdman L., Rickard M., Lorenzo A.J., Hung A.J., Farcas M., Goldenberg L. (2021). Standardized Reporting of Machine Learning Applications in Urology: The STREAM-URO Framework. Eur Urol Focus..

[24] Chipollini J., De la Rosa A.H., Azizi M., Shayegan B., Zorn K.C., Spiess P.E. (2019). Patient presentation, differential diagnosis, and management of penile lesions. Can Urol Assoc. J..

[25] Teichman J.M.H., Mannas M., Elston D.M. (2018). Noninfectious Penile Lesions. Am. Fam. Physician.

[26] Crispen P.L., Mydlo J.H. (2010). Penile intraepithelial neoplasia and other premalignant lesions of the penis. Urol. Clin. N. Am..

[27] Menzies S.W., Sinz C., Menzies M., Lo S.N., Yolland W., Lingohr J., Razmara M., Tschandl P., Guitera P., Scolyer R.A. (2023). Comparison of humans versus mobile phone-powered artificial intelligence for the diagnosis and management of pigmented skin cancer in secondary care: A multicentre, prospective, diagnostic, clinical trial. Lancet Digit. Health.

[28] Jobson D., Mar V., Freckelton I. (2022). Legal and ethical considerations of artificial intelligence in skin cancer diagnosis. Australas. J. Dermatol..

[29] Sangers T.E., Kittler H., Blum A., Braun R.P., Barata C., Cartocci A., Combalia M., Esdaile B., Guitera P., Haenssle H.A. (2024). Position statement of the EADV Artificial Intelligence (AI) Task Force on AI-assisted smartphone apps and web-based services for skin disease. J. Eur. Acad. Dermatol. Venereol..

